# Editorial: The future of farm animal welfare science: selected papers from the 9th International Conference on the Welfare Assessment of Animals at Farm Level (WAFL)

**DOI:** 10.3389/fvets.2026.1791497

**Published:** 2026-02-06

**Authors:** Tina Widowski, Jen-Yun Chou, Maria José Hötzel, Gabriela Olmos Antillón

**Affiliations:** 1 Campbell Centre for the Study of Animal Welfare, University of Guelph, Guelph, ON, Canada; 2 Ethology and Welfare, Prairie Swine Centre, Saskatoon, SK, Canada; 3 Universidade Federal de Santa Catarina, Florianópolis, Brazil; 4 Veterinary Epidemiological Unit, Department of Clinical Sciences, Swedish University of Agricultural Sciences, Uppsala, Sweden

**Keywords:** animal-based measures, evidence-based policy, human–animal interactions, on-farm assessment, precision livestock farming, welfare benchmarking, welfare governance, welfare indicators

The welfare of farm animals is central to the sustainability of food production. Valid and practical methods for assessing animal welfare on farms, during transport, and at slaughter are key to ensuring that their welfare is protected and are increasingly required for the production and marketing of animal products worldwide. The development of assessment methods and sound animal welfare policy should be based on a solid foundation in animal welfare science, as well as an understanding of the social factors that influence improvements in farm animal welfare. Launched in 1999, the Welfare Assessment of Animals at Farm Level (WAFL) is an international conference held every 3 years that focuses specifically on these issues (1). This Research Topic features papers submitted to the 9th WAFL International Conference, which was held in Florence, Italy, on August 30–31, 2024.

One major theme at WAFL 2024 was “New Frontiers in the Assessment of Animal Welfare On-Farm.” Under this topic, Czycholl et al. conducted a systematic review of the scientific literature on fixed-list Qualitative Behavior Assessment (FL-QBA), a whole-animal approach to capturing affective expression that complements conventional welfare indicators. They identified 193 peer-reviewed FL-QBA papers and showed how the method has diversified. The review provides a deep methodological examination of these studies by identifying wide variations in fixed-list development, observer training/reliability, observation format and duration, VAS design, and analytics, along with inconsistent reporting, making cross-study comparisons and standard-setting challenging. This paper is a must-read for researchers planning on using the FL-QBA approach. Day et al. contributed a novel, data-driven approach to solving one of the core challenges in welfare assessment: selecting efficient yet informative sets of welfare indicators under practical constraints. By developing and testing an optimisation algorithm grounded in risk assessment frameworks, the authors demonstrate how indicator selection can be tailored across species and contexts while maintaining scientific robustness and practical feasibility. Markland, et al. addressed a central challenge in farm-level welfare assessment by examining how damaging and aggressive behaviors in pigs relate to the development of ear, tail, and flank lesions across production stages. By identifying stage-specific associations and the thresholds at which behaviors result in lesions, the study highlights the complexity of using behavioral indicators as early warning signals of compromised welfare in intensive pig production systems.

Although the WAFL conference focuses on the welfare of animals, the humans surrounding the animals are equally crucial and influence animal welfare outcomes. From another of the conference themes, “Humans in the Loop,” Tamminen, et al. explored how dairy farmers and other stakeholders perceive and negotiate the meaning, usefulness, and feasibility of automated welfare monitoring tools. The findings reveal tensions between expectations, agency, and infrastructural constraints, underscoring the importance of co-development, shared understanding, and long-term advisory support for the successful implementation of welfare technologies on farms. Olmos Antillón, et al. extended welfare science beyond biological assessment by examining how the perceived social value of animals shapes veterinary antimicrobial use across species and countries. Using social practice theory, the authors reveal species-based hierarchies of care that influence diagnostic rigor, treatment decisions, and follow-up practices, with important implications for antimicrobial stewardship, animal welfare, and public health. Lundmark Hedman et al. provided a look at welfare assurance schemes by comparing how Swedish official animal welfare inspectors and private welfare auditors experienced their own on-farm inspections. Using a national questionnaire, the study showed that both groups generally enjoyed the work and emphasized good dialogue and uniform assessments as critical for effective inspection, while also acknowledging the difficulty of maintaining uniformity in real-world settings. Official inspectors more often reported unpleasant situations and a more skeptical view of animal keepers' welfare knowledge and conditions, whereas private auditors more often encountered keepers who appeared prepared and relaxed. The paper highlights better collaboration, inspector training, and information sharing as practical ways to strengthen animal welfare governance.

WAFL 2024 also explored the “Future of Animal Welfare through Policy and Science.” Data-driven decision-making tools, such as Benefit Cost Analysis (BCA), are often used by policymakers to address aspects of human health and welfare or the environment. These approaches require monetizing and weighing all costs and benefits of a policy, even intangible ones like quality of life or air quality. Here, Fischer provides a perspective on how animal welfare scientists and veterinarians can and should collaborate with economists, policy analysts, and philosophers to develop valuation methods that best represent animal welfare in policy decisions. Foris et al. critically examine the role of animal welfare scientists in shaping the development, validation, and implementation of AI-based welfare assessment tools. Framed within the One Welfare concept, the authors discuss scientific, ethical, and practical uncertainties, emphasizing that meaningful welfare gains depend on interdisciplinary collaboration and careful consideration of embedded values and real-world impacts. A vast variety of measures have been developed and tested for use in animal welfare assessment protocols under commercial conditions, and there is a need for standardization and harmonization if they are to be used for benchmarking and comparing the status of farm animal welfare across regions and settings. In their literature review, de Jong et al. reviewed over 250 scientific articles on farm animal welfare assessment protocols published between 2013 and 2023 to determine how measures represented the five welfare domains and aligned with the European Food Safety Authority (EFSA) welfare consequences. From this, the authors identified strengths, weaknesses, and areas in need of future development, especially for understudied species.

How to apply science to practice is another focus of WAFL. Tadich et al. addresses an underrepresented group of animals and the limited research attention it has received. The paper investigates the physiological responses of mules to increasing load weights under controlled conditions. By identifying workload thresholds that elicit measurable stress responses while remaining within adaptive limits, the paper provides much-needed empirical evidence to inform welfare-relevant guidelines for the management and use of working equids. Phelipon et al. examine whether the fundamental welfare needs of horses—access to forage, freedom of movement, and social interactions (the “3Fs”)—are respected in high-level sport horses and how their provision relates to behavioral and physical welfare indicators. Based on on-farm assessments of internationally competing horses, the study identifies consistent associations between fewer restrictions and improved welfare outcomes, but high variability in the implementation of the 3Fs. The authors conclude that compliance with core welfare principles is both feasible and beneficial in elite horse sport contexts.

Cow–calf contact systems (CCS) are gaining interest as more “natural” alternatives. Foster CCS may be a compromise, as selected cows nurse multiple calves and are usually not milked during this period. Zipp et al. conducted a study on a large organic commercial dairy farm in Germany to evaluate some aspects of fostering cows' welfare. Foster cows experienced a higher risk of teat lesions, especially during the mid-nursing period, with no clear negative effects on teat skin dryness, overall body condition, or fertility. Teat lesions are a welfare concern because they can be painful, increase infection risk, and indicate aversive cow–calf interactions. Rademann et al. used a Welfare Quality^®^ assessment to compare the welfare of the calves and heifers raised under CCC systems with those experiencing early separation on commercial dairy farms. Farms practicing CCC achieved higher welfare scores across behavioral, management, and resource-based indicators, especially for positive emotional state, appropriate behavior, space allowance, and access to pasture, whereas differences in physical health were less pronounced. These studies illustrate how standardized, animal-based welfare assessments can distinguish welfare outcomes between management systems and provide evidence relevant to on-farm decision-making and policy.

Overall, these papers highlight both the advances in animal welfare assessment science and the applications on farms and in governance. Together, they reinforce WAFL's core message: rigorous methodology, transparent implementation and positive human relationship are all essential to delivering measurable and credible improvements in farm animal welfare.
